# BAG3-related myopathy, polyneuropathy and cardiomyopathy with long QT syndrome

**DOI:** 10.1007/s10974-015-9431-3

**Published:** 2015-11-06

**Authors:** Anna Kostera-Pruszczyk, Małgorzata Suszek, Rafał Płoski, Maria Franaszczyk, Anna Potulska-Chromik, Piotr Pruszczyk, Elżbieta Sadurska, Justyna Karolczak, Anna M. Kamińska, Maria Jolanta Rędowicz

**Affiliations:** Department of Neurology, Medical University of Warsaw, 1a Banacha St., 02-097 Warsaw, Poland; Department of Biochemistry, Nencki Institute of Experimental Biology, 3 Pasteur St., 02-093 Warsaw, Poland; Department of Medical Genetics, Medical University of Warsaw, 3c Pawińskiego St., 02-106 Warsaw, Poland; Laboratory of Molecular Biology, Institute of Cardiology, 42 Alpejska St., 04-628 Warsaw, Poland; Department of Internal Medicine and Cardiology with the Center for Diagnosis and Treatment of Venous Thromboembolism, Medical University of Warsaw, 4 Lindleya St., 02-005 Warsaw, Poland; Department of Pediatric Cardiology, Medical University of Lublin, Chodźki 2, 20-093 Lublin, Poland

**Keywords:** BAG3, HSP70, Long Q-T syndrome myopathy, Sarcomere, Z-disc

## Abstract

BAG3 belongs to BAG family of molecular chaperone regulators interacting with HSP70 and anti-apoptotic protein Bcl-2. It is ubiquitously expressed with strong expression in skeletal and cardiac muscle, and is involved in a panoply of cellular processes. Mutations in *BAG3* and aberrations in its expression cause fulminant myopathies, presenting with progressive limb and axial muscle weakness, and respiratory insufficiency and neuropathy. Herein, we report a sporadic case of a 15-years old girl with symptoms of myopathy, demyelinating polyneuropathy and asymptomatic long QT syndrome. Genetic testing demonstrated heterozygous mutation Pro209Leu (c.626C > T) in exon 3 of *BAG3* gene causing severe myopathy and neuropathy, often associated with restrictive cardiomyopathy. We did not find a mutation in any known LQT syndrome genes. Analysis of muscle biopsy revealed profound disintegration of Z-discs with extensive accumulation of granular debris and large inclusions within fibers. We demonstrated profound alterations in BAG3 distribution as the protein localized to long filamentous structures present across the fibers that were positively stained not only for α-actinin but also for desmin and filamin indicating that those disintegrated Z-disc regions contained also other sarcomeric proteins. The mutation caused a decrease in the content of BAG3 and HSP70, and also of α-actinin desmin, filamin and fast myosin heavy chain, confirming its severe effect on the muscle fiber morphology and thus function. We provide further evidence that BAG3 is associated with Z-disc maintenance, and the Pro209Leu mutation may occur worldwide. We also provide a summary of cases associated with this mutation reported so far.

## Introduction

Myofibrillar myopathies (MFMs) are a group of genetically heterogeneous disorders characterized by myofibrillar degeneration. Accumulation of numerous Z-disc proteins, such as αB-crystallin, desmin, or myotilin is a morphological hallmark of MFM. Most MFM patients present in early adulthood with progressive limb muscle weakness or cardiomyopathy (Selcen 2011; Olivé et al. 2013; Fichna et al. 2014). Although distal muscles are frequently involved in MFM patients and may mimic neuropathy, sensory-motor polyneuropathy is a rare feature. Childhood-onset MFMs are usually associated with rapidly progressing cardiac dysfunction, however, long QT (LQT) syndrome was reported only once in a MFM patient with desmin gene mutation and variants in two LQT genes, SCN5A and KCNH2 (Sung et al. 2010).

So far several causative mutations of the MFM were identified in numerous genes, among them was BAG3 also known as Bcl-2-associated athanogene 3 or Bcl-2-binding protein Bis. It belongs to BAG family of multi-domain proteins playing a role of regulators of molecular chaperones (Rosati et al. 2011). BAG3 (MW ~74 kDa) consists of N-terminal bi-partite WW-domain involved in protein–protein interactions that is followed by a proline-rich domain (PXXP), also mediating binding to other proteins, and C-terminal BAG domain, which binds to heat shock protein Hsp 70 and the anti-apoptotic protein Bcl-2 (Doong et al. 2003; Odgerel et al. 2010; Rosati et al. 2011). Interaction of BAG3 with Hsp70 modulates its chaperone activity (Doong et al. 2003; Rosati et al. 2011). Through these interactions BAG3 is involved in a panoply of cellular processes such as development, apoptosis, autophagy as well as cytoskeleton organization, cell adhesion and motility (Rosati et al. 2011). *BAG3* is ubiquitously expressed in all the tissues with the strong expression in skeletal and cardiac muscle as well as in cancer cells (Homma et al. 2006; Iwasaki et al. 2007). In cardiomyocytes BAG3 was also found to regulate the structural stability of F-actin through the actin capping protein, CapZβ1, by promoting association between Hsc70 and CapZβ1. It is therefore proposed that BAG3 and Hsc70 play important role in stabilizing myofibril structure and inhibiting myofibrillar degeneration in response to mechanical stress (Hishiya et al. 2010). It has been also shown that BAG-3 is important for mobilization of filamin from the Z disk, and for subsequent ubiquitine-dependent degradation of filamin (Arndt et al. 2010).

*Bag3* deficiency in mice resulted in fulminant myopathy and early lethality (Homma et al. 2006). Also, its expression is induced during cardiomyoblast differentiation and it can modulate myogenin expression (De Marco et al. 2011). Several *BAG3* mutations and polymorphisms were reported so far in humans. Heterozygous Pro209Leu (*626C* > *T*) mutation is the only one associated with neuromuscular phenotype (see Table 1). It was first reported in three patients with progressive limb and axial muscle weakness, who also developed cardiomyopathy and severe respiratory insufficiency in their teens; two had rigid spine and one peripheral neuropathy (Selcen et al. 2009). The same mutation was found in several other further patients worldwide with severe and variable phenotypes (Jaffer et al. 2011; Konersman et al. 2015). Functional studies revealed that BAG3 mutations impaired the Z-disc assembly and increased the sensitivity to stress-induced apoptosis (Selcen et al. 2009; Arimura et al. 2011; Konersman et al. 2015). Also, several other mutations were found in early and late onset of dilated cardiomyopathy (DCM) patients of different ethnicity (Norton et al. 2011; Chami et al. 2014; Franaszczyk et al. 2014).

**Table 1:** Summary of reported cases with Pro209Leu (c.626C > T) *BAG3* mutations

Report	Proband (age at onset presenting feature)	Cardiomyopathy	QTc (s)	Skeletal myopathy	Neuropathy	Other features	Outcome at last observation
Current report	Female (12 years) Toe-walking Foot deformity	Restrictive (subclinical at 15 years)	Mean 478 ms Max 574 ms	Subclinical	Axono-demyelinating neuropathy; nerve biopsy not done	Rigid spine, Contractures, FVC 87 %	Ambulant, no cardiac symptoms at 15 years
Jaffer et al. (2012)	Female (1.5 years) Toe-walking	Restrictive (heart transplantation at 13 years)	Not reported	Proximo-distal weakness and atrophy	Giant axonal neuropathy	Rigid spine, elbow/finger flexion contractures	Lost ambulation at 16 years
	Male (early childhood) Toe-walking, later steppage gait	Restrictive (8 years)	Not reported	Proximo-distal weakness and atrophy	Giant axonal neuropathy	Rigid spine, Pes carinatum; FVC 53 % (13 years)	Ambulant (13.5 years) Non-invasive ventilation
	Male (11 months) Toe-walking “clumsy”	Restrictive	Not reported	Severe proximo-distal weakness	Giant axonal neuropathy	Rigid spine scoliosis, Achilles tendon tightness FVC 50 % (11 years)	Died of cardiorespiratory failure at 12^9^/_12_)
	Female (first decade), toe-walking, mobility impairment	Restrictive	Not reported	Mild	Axonal neuropathy	*pes cavus*, tight Achilles tendons, scoliosis (Cobb angle 104^◦^); FVC 46 %	Non-ambulant at 14 years; non-invasive nocturnal ventilation
	Female (older sister) similarly affected, father died at 30 years - similar presentation						
Odegrel et al. (2010)	Male (9 years)	Restrictive/hypertrophic (9 years)	Not reported	Yes	Giant axonal neuropathy	Transmission from asymptomatic father with somatic mosaicism	Sudden death at 9 years
	Male (12 years)	Restrictive/ hypertrophic Heart transplantation (14 years)	Not reported	Severe	Giant axonal neuropathy		Wheelchair at 14 years Ventilator dependent at 29 years
	Female (12 years). *pes cavus*, muscle weakness; cardiomyopathy	Restrictive/ Hypertrophic	Not reported	Neck and distal muscle weakness	Giant axonal neuropathy		Died at 20 years of cardiac and respiratory failure
	Male (5 years), problem running	Restrictive/hypertrophic (10 years); Heart transplantation (13 years)	Not reported	Proximal muscle weakness	Giant axonal neuropathy		Ventilator dependent at 13 years Died at 15 years
Lee et al. (2012)	Female (6 years), clumsy walking	Restrictive/ hypertrophic	450–570 ms	Mild	Axonal neuropathy	Rigid spine and scoliosis; contractures and foot deformity	Ambulant at 12 years
Konersman et al. (2015)	Male (8 years), heart transplant	Restrictive, orthotopic heart transplant at 8 years	Not reported	Severe	Sensorimotor polyneuropathy	Imbalance, spinal rigidity	19 years, intermittent ventilation at day and continuous at night via tracheostomy; feet and ankles paralyzed, ambulant with a walker across short distances

In the present study, we report the case of 15-year old girl with Pro209Leu in *BAG3* with MFM, sensory-motor polyneuropathy and long QT syndrome. We also show the effect of the mutation on the muscle fiber organization and the amount of both BAG3 and HSP70 as well as of other sarcomeric proteins. Additionally, we provide summary of the effect of Pro209Leu mutation on *BAG3*-related myopathies characterized so far.

## Materials and methods

### Genetic analyses

DNA was extracted from peripheral blood of the proband using standard methods. Screening for BAG3 mutations was performed by direct sequencing using ABI 3130 Genetic Analyzer (from Applied Biosystems, USA). Whole Exome Sequencing (WES) was performed on HiSeq 1500 (Illumina, USA) as previously described (Ploski et al. 2014).

### Muscle biopsy

The open muscle biopsy of the proband quadriceps was performed, and the muscle specimens was processed for further analyses and compared with normal muscle biopsy. The study was performed in compliance with the national legislation and the Code of the Ethical Principles for Medical Research Involving Human Subjects of the World Medical Association.

### Light microscopy

Part of the biopsied tissue, to be used for light microscopy, was frozen in isopentane cooled in liquid nitrogen, cut on a cryomicrotome with a slice thickness of 8 µm and stained with the routine battery of histological and histochemical methods.

### Electron microscopy analysis of the muscle biopsy

For electron microscopy, a part of the muscle specimen was fixed in glutaraldehyde, post fixed in osmium tetroxide before embedding in Spurr embedding medium (Electron Microscopy Sciences, USA). Ultrathin sections of the selected areas were stained with uranyl acetate and counterstained with lead citrate. The samples were viewed with JEM 1200 EX2 electron microscope. Quantitative analysis of disorganized areas of the proband’s muscle fiber was performed on 12 electronmicrographs covering different regions of the muscle using ImageJ software.

### Antibodies and fluorescent markers

Rabbit polyclonal antibody raised against a recombinant fragment corresponding to the C terminal 196 amino acids of human BAG3 and mouse monoclonal antibody raised against human sarcomeric α-actinin were from Abcam (UK). The following monoclonal antibodies were also used: anti-HSP70 from Enzo Life Sciences (USA), anti-desmin from BD Pharmingen (USA), anti-chicken gizzard filamin (F1888) from Sigma–Aldrich (USA), anti-rabbit fast myosin heavy chain (Abcam, UK) and anti-GAPDH (glyceraldehyde 3-phosphodehydrogenase) from Merck Millipore (USA). For immunocytochemistry studies, the following secondary antibodies were used: goat anti-rabbit IgG conjugated with AlexaFluor-488 or rabbit anti-mouse IgG conjugated with AlexaFluor-546 (from Molecular Probes, USA and Jackson ImmunoResearch Europe Ltd., UK, respectively). DAPI (a dye staining chromatin) was applied in the Vectashield mounting medium (Vector Labs, USA). For western blot analysis the following secondary antibodies were used: anti-rabbit or anti-mouse secondary antibodies conjugated with horse radish peroxidase (Merck Millipore, USA).

### Immunolocalization studies

Distribution of BAG3, α-actinin (the marker of Z-disc), filamin and desmin in muscle biopsies obtained from the patient and healthy subject was examined by indirect immunohistochemistry. Muscle cross-sections were fixed in 4 % paraformaldehyde for 10 min. The fixed specimens were thoroughly washed in phosphate-buffered saline (PBS) and treated for 30 min with a solution of 5 % normal goat serum and 0.2 % Triton X-100 in PBS. Subsequently, muscle sections were incubated overnight at 4 °C with the anti-BAG3 antibody at a dilution of 1:200 and followed by incubation with Alexa 488 conjugated anti-rabbit secondary antibodies at a dilution of 1:200 for 60 min RT. For simultaneous assessment of the distribution of other sarcomeric proteins, muscle sections were incubated with the α-actinin antibody at a dilution of 1:50, desmin antibody (at 1:100) and anti-filamin antibody (1:100), and then with secondary anti-mouse antibody at a dilution 1:500 for 60 min RT. To mount the slides and visualize the nuclei, the Vectashield mounting medium with DAPI was applied. The specimens were visualized using Zeiss LSM 780 spectral confocal microscope equipped with an HCX PL APO 40x/1.4 Oil DIC M27 objective. Optical Sects. (2048 pixels × 2048 pixels × 8 bits/pixel) were collected at 0.30 µm z-spacing. In concomitant staining with two or three fluorophores, special care was taken to control for any possible cross-talk of the detection systems. We carefully adjusted the spectral ranges of the detectors and always scanned the images sequentially. For negative controls, the primary antibody was omitted.

### Immunoblot analyses

Control and patient’s muscles were homogenized in 10 volumes over the muscle mass of ice-cold PBS supplemented with 1 mM phenylmethylsulfonyl fluoride (PMSF) and Complete protease inhibitor cocktail (Roche Diagnostics GmbH, Germany). To evaluate the amount of BAG3, HSP70, α-actinin, desmin, filamin and fast isoform of myosin heavy chain (fMHC), both total muscle homogenates and soluble fractions (obtained after centrifugation of homogenates at 1800×*g*) were examined. Equal volumes of the samples were separated on 10 % SDS-PAGE, and transferred to a nitrocellulose membrane. The immunoreactive bands were detected by incubation with antibody against BAG3 (at 1:2000 dilution), HSP70 (1:1000), α-actinin (1:500), desmin (1:1000), fMHC (1:1000) and GAPDH (1:10000 dilution) followed by the incubation with respective secondary antibodies conjugated with horse radish peroxidase. The reaction was developed using ECL according to the manufacturer’s instructions (Pierce, USA). Developed blots were photographed using a G:Box system equipped with the GeneSnap software.

## Results

### Case description

**Fig. 1:**
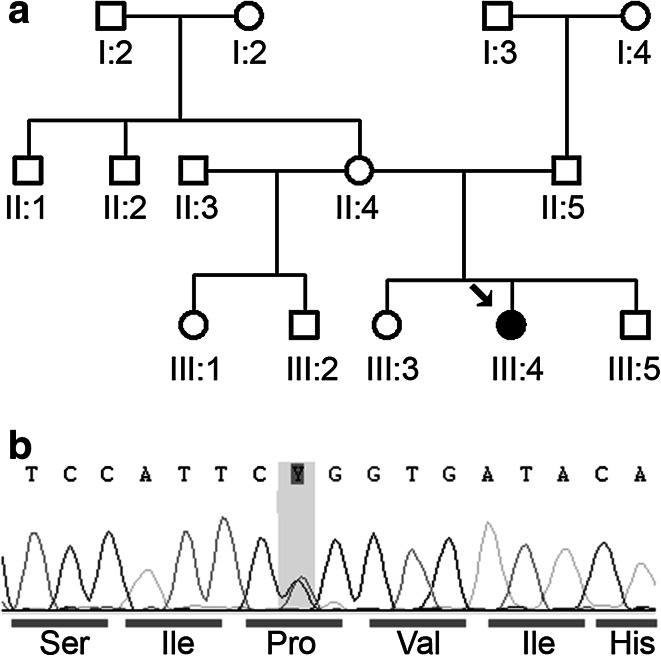
Identification of Pro209Leu mutation in *BAG3*. **a** Pedigree diagram of the proband’s family. The *arrow points* to the proband (*black symbol*); *open symbols* indicate unaffected family members. **b** Chromatograms illustrating identification of Pro209Leu mutation in the proband

We report a 15-year old Caucasian girl, oldest child of unaffected, non-consanguineous parents; she has two unaffected sisters (Fig. 1a). Developmental milestones were normal, she walked unsupported at 12 months of age but at 8 years she started to toe-walk and developed foot deformity. She had no cardiac signs or symptoms. Long QT was first seen on routine ECG before foot surgery when she was 11. At 12 years she presented with bilateral *pes cavus* deformity, deep tendon reflexes in lower extremities were absent, vibration sense was reduced up to the ankles. She was toe-walking but had no other motor impairment. Nerve conduction studies revealed axonal-demyelinating sensory-motor polyneuropathy with conduction velocity (cv) in median/ulnar motor nerves 38 m/s. Echocardiography at that time was not relevant. The level of creatine kinase (CK) was elevated to 1.5 times upper limit of normal. At 13 years she was diagnosed with restrictive cardiomyopathy. She had rigidity of cervical and thoracic spine and contractures at hips, knees and ankles. Muscle biopsy confirmed myofibrillar myopathy. She was last seen at 15 years with asymptomatic long QT. Her pulmonary function was normal [forced vital capacity (FVC) 87 %]. Spinal rigidity and contractures further limited her mobility but she was ambulant. The blood sample was subjected to direct sequencing for *BAG3* and heterozygous mutation Pro209Leu (c.626C > T) in exon 3 was revealed (see Fig. 1b). Proband’s mother, father and siblings do not carry this mutation and do not present any muscle dysfunction (Fig. 1a).

### Whole exome sequencing (WES)

In order to search for other genetic defects which could cause LQT syndrome in the proband, we analyzed the WES results focusing on the following genes linked with LQT syndrome according to HGMD and/or OMIM: *ACN9, AKAP9, ALG10, ALG10B, ANK2, CACNA1C, CACNA2D1, CACNB2, CALM1, CALM2, CALM3, CAV3, FRS2, KCNE1, KCNE2, KCNE2, KCNE3, KCNH2, KCNJ2, KCNJ5, KCNQ1, RYR2, SCN4B, SCN5A, SEC61G, SNTA1, TBXAS1* and *TYMS.*

The mean coverage of these loci in our WES analysis was 60; 96,9 % of the coding sequence of this target had coverage >10 and 84,8 % - coverage >20. We filtered the data to include rare variants (<1 % in available databases such as EXAC, ESP 6500, 1000 Genomes and our own collection of >400 exomes) affecting the coding sequence or splice sites. The only variant found was a missense change in the *SCN5A* gene (Chr3:38598723 C > T, NM_000335.4:p.Gly1432Glu), which was not reported previously. Using Sanger sequencing we typed proband’s parents and established that the variant came from the mother. Since she was free from LQT syndrome, we concluded that *SCN5A* p.Gly1432Glu is not pathogenic.

### Morphological analysis of the proband’s muscle

**Fig. 2:**
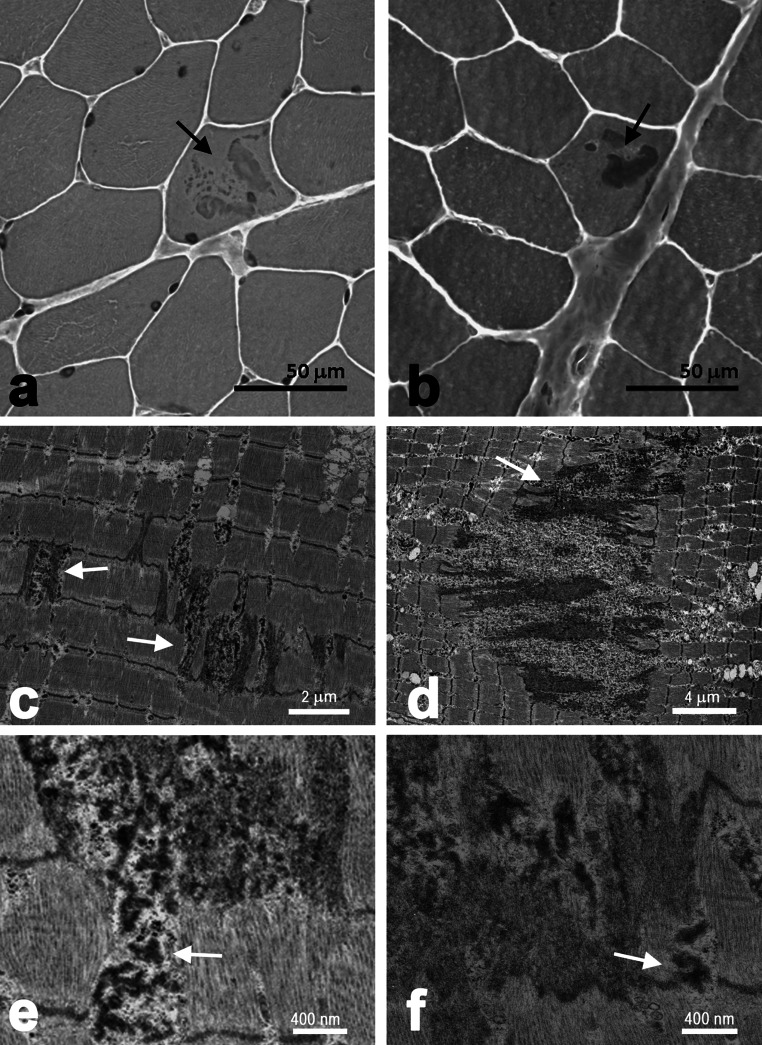
Analysis of morphology of the proband’s muscle. **a**, **b** Hematoxylin/eosin and Trichrome stainings, respectively, of the proband’s muscle. *Arrows* *point* to large deposits within the muscle fiber. **c**–**f** Electron microscopy images of the longitudinal sections of the proband’s muscle. Explanation for the *arrows* in **c**–**f** in the “Results” section. *Bars* in **a**–**f**, as indicated on the images

Hematoxylin/eosin staining of muscle transverse sections (Fig. 2a) revealed the presence of large deposits within some fibers (marked by an arrow). The presence of large deposits was also shown using Trichrome staining (Fig. 2b, arrow). Noteworthy, no major variability in the size of the muscle fibers was visible.

Electron microscopy of longitudinal ultrathin sections showed dramatic changes in the fiber organization (Fig. 2 c–f). Z-disc streaming (Fig. 2c, arrows) and disorganization of myofibrils as well as large areas of sarcomeric disorganization with accumulation of polymorphic dense structures (Fig. 2d, arrow) were present. Also, focal changes containing granulomatous material (Fig. 2e, arrow) and abnormal regions with the presence of multiple dense bodies (Fig. 2f, arrow) were visible. Qualitative analysis of disorganized areas revealed that their size significantly varied. We observed regions as small as about less than 1 μm^2^ (for example the one marked with left arrow in Fig. 2c was 3 μm^2^) and as large as about 176 μm^2^ (for example that in Fig. 2d). The size of long axis of a largest disorganized area examined reached the value of about 20 μm.

### Localization of BAG3 and α-actinin in the proband’s muscle

**Fig. 3:**
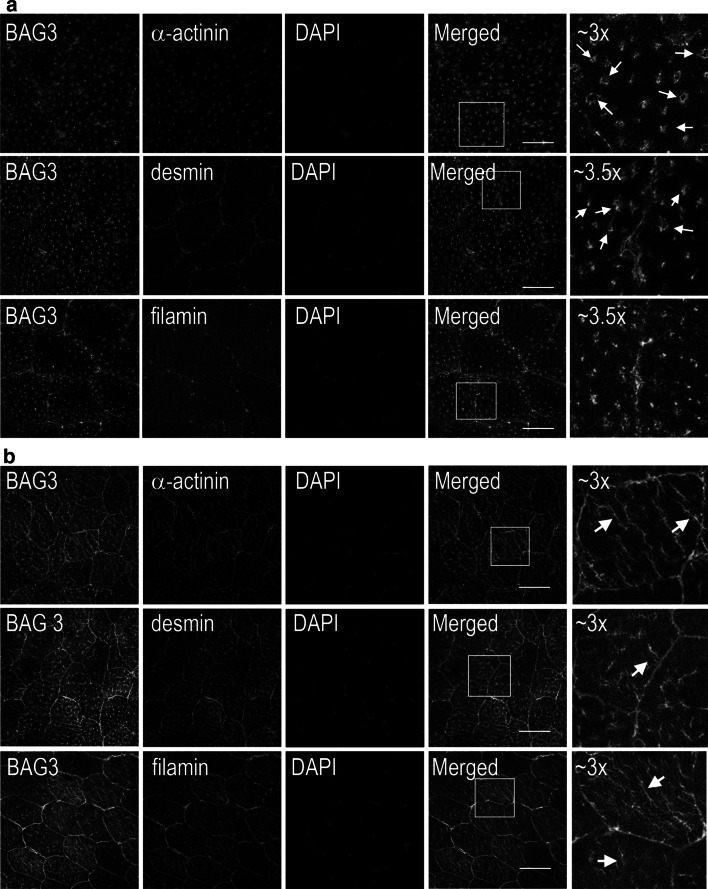
Distribution of BAG3 in human muscle fibers. Transverse sections of the muscle biopsy of the healthy subject (**a**) and proband (**b**) were stained with anti-BAG3 antibody (*in green*) as well as with anti-α-actinin, anti-desmin and anti-filamin antibodies (all in *red*). Nuclei were stained with DAPI (in *blue*). Far* right panels*, ~3 to ×3.5 magnification of the areas marked in **a** and **b**. These are 0.3-μm images of the center of transverses muscle section obtained with a Zeiss confocal microscope. *Arrows* in** a**, point to the presence of BAG3 within the Z-disc in control muscle; and in **b**, point to its association with severely affected Z-discs containing α-actinin, desmin or filamin. Other details as described in Materials and Methods section. *Bars* 50 μm. (Color figure online)

To test whether the Pro209Leu point mutation within *BAG3* affects its distribution in the patient’s muscle, immunostaining of the control and proband quadriceps muscle biopsies was performed (Fig. 3a, b). Also, localization of α-actinin, the protein marker of Z-disc as well as of other sarcomeric proteins, namely desmin and filamin was assessed in the proband’s and control muscles (Fig. 3a, b).

In the transverse section of the control muscle BAG3 was present mainly within the Z-disc where it colocalized with α-actinin (Fig. 3a, upper panels, arrows). It was also adjacent to desmin, which was surrounding BAG3-stained Z-discs (Fig. 3a, middle panels, arrows). BAG3 did not co-localize with filamin but it seemed to be embedded in filamin meshwork present throughout the fiber (Fig. 3a, lower panels).

However, this defined BAG3 distribution was severely impaired in vast majority of fibers of the patient’s muscle (Fig. 3b). BAG3 was seen in long structures present within the fibers with the variable length, ranging from about 5 μm up to seemingly over 30 μm (Fig. 3b, arrows). These elongated structures were also positively immunostained with anti-α-actinin, anti-desmin and anti-filamin antibodies showing that mutated BAG3 was associated with severely disintegrated Z-discs where other sarcomeric proteins were recruited.

### Assessing BAG3 expression level in the proband’s muscle

**Fig. 4:**
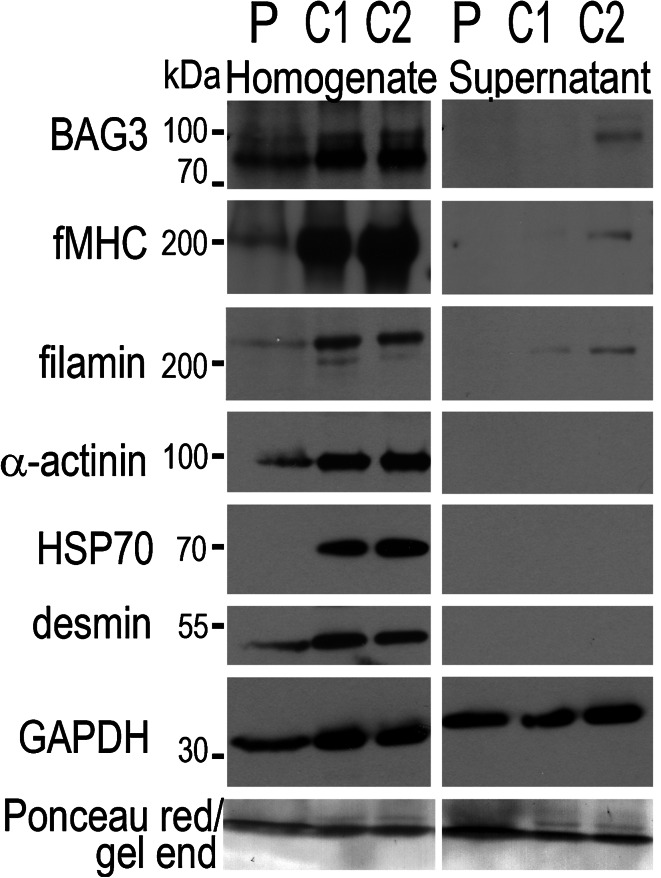
Analysis of BAG3 and Hsp70 content in the proband (P) and healthy subjects (C1 and C2) muscle by immunoblotting. Equal volumes of muscle homogenates and soluble fractions were subjected to SDS polyacrylamide gel electrophoresis, blotted on nitrocellulose membrane and probed with anti-BAG3, anti-HSP70, anti-α-actinin, anti-desmin, anti-filamin and anti-fast myosin heavy chain (fMHC) antibodies as described in Materials and Methods. Probing with anti-GAPDH antibody served as the internal loading control

To examine the amount of BAG3 in the patient’s muscle, we performed immunoblotting with anti-BAG3 antibody (Fig. 4). The overall BAG3 content (with respect to the amount of GAPDH) in the patient’s homogenate (P) was reduced when compared to the control samples (C1 and C2). We also checked whether and how BAG3 mutation affects the content of HSP70 as well as of other sarcomeric proteins, α-actinin, desmin, filamin and fMHC. Of note, we also probed the samples for slow myosin heavy chain but did not detect any signal (not shown). As shown in Fig. 4, in muscle homogenates the intensity of the bands corresponding to those proteins was substantially decreased. As expected, we did not see these sarcomeric proteins in soluble fraction of control muscles. The same was observed for patient’s sample thus suggesting that mutated BAG3 (and other affected partners) could form insoluble aggregates or if they were soluble they were fragmented into small peptides that migrated out of the gel. The latter seems to be confirmed by remarkably more intensive Ponceau red staining at the gel end that was visible in the patients’s samples (Fig. 4).

## Discussion

We report the complex phenotype with LQT syndrome of a girl with Pro209Leu *BAG3* mutation and provide further evidence for association of mutated BAG3 with disarrayed Z-disc within the muscle biopsy of the patient. We also show that this mutation affects the amount of HSP70 and other sarcomeric proteins, including α-actinin, the Z-disc marker.

*BAG3* mutations are rare and were not found in analysis of cohorts of 21 and 35 families with myofibrillar myopathies (Olive et al. 2011; Vattemi et al. 2011). Severe neuromuscular phenotype is associated with Pro209Leu point heterozygous mutation that appears sporadically and probably de novo (see Table 1). So far only two families with two affected siblings were reported. In the first family, father had somatic mosaicism (Odgerel et al. 2010) and in the second, father was affected by hearsay (Jaffer et al. 2012). Pro209Leu leads not only to limb muscle dysfunction but also to early respiratory insufficiency or cardiomyopathy requiring heart transplantation by 20th birthday (Odgerel et al. 2010; Jaffer et al. 2012; Konersman et al. 2015). Interestingly, long QT symptoms, severe muscle weakness and hypertrophic cardiomyopathy were present in a Chinese patient with both Pro209Leu mutation and Arg258Trp (*772C* > *T*) substitution (Lee et al. 2012). In this family, proband’s father who was a carrier of the Arg258Trp mutation had long QT but no other phenotypic features (Lee et al. 2012). Several other BAG3 mutations were found in early- and late onset dilated cardiomyopathy (DCM) patients without the obvious skeletal muscle phenotype (Franaszczyk et al. 2014).

Our patient demonstrated long QT in 90 % of recorded tracings on Holter ECG with maximal QTc of 578 ms. We were not able to find a cause of her long QT other than *BAG3* mutation. Although there is possibility that the patient is carrying more than one mutation responsible for a cardiac phenotype, no mutation in any LQT-related genes was found.

The observed herein changes within the fiber are in line with other studies describing skeletal muscles of the myofibrillar myopathy patients with the Pro209Leu mutation (Selcen et al. 2009; Jaffer et al. 2012; Odgerel et al. 2010; Lee et al. 2012). Interestingly, majority of *BAG3* mutations identified so far, namely Pro209Leu (causing myofibrillar myopathy), Arg218Trp (causing dilated cardiomyopathy) as well as one of four novel mutations associated with fulminant dilated cardiomyopathy in Polish patient (a large deletion of 17,990 bp removing *BAG3* exons 3–4) are located within the exon 3 indicating importance of this region for the protein activity (Arimura et al. 2011; Selcen et al. 2009; Franaszczyk et al. 2014). Moreover, there are several phoshorylatable residues including Ser194, 195, 198 and 207, and Tyr205 in the region preceding Pro209 residue (http://www.phosphosite.org/proteinAction.do?id=5352&showAllSites=true). Thus this part of the molecule could undergo conformational changes and be crucial for the proper BAG3 conformation/folding. Hence, replacement of this highly conserved proline residue with leucine might affect the overall BAG3 tertiary structure and possibly its stability, and interaction with its partners engaged in Z-disc assembly as it was originally proposed by Selcen et al. (2009). It is also plausible that the mutation impairs chaperone activity of heat shock proteins despite that it affects the BAG domain not involved in interaction with HSP70 (Rosati et al. 2011).

Arimura et al. (2011) suggested that Pro209Leu mutation could also disturb the multinucleation during the myoblast differentiation into skeletal muscle myotubes. In our and other studies, it was shown that the mutation was causing severe disintegration of the Z-disc (Selcen et al. 2009, Odgerel et al. 2010, Lee et al. 2012). We have demonstrated that mutated BAG3 in the proband’s muscle fibers still localizes to the Z-disc marker, α-actinin, and the disorganized fiber areas contain other sarcomeric proteins such as desmin and filamin, indicating its association with Z-disc despite its profound disintegration. Our observation that the level of HSP70 was practically undetectable in the proband’s muscle and the content of α-actinin, desmin, filamin and fast myosin heavy chain was substantially decreased is therefore in line with the notion that properly functioning BAG3 is important for proper organization of the myofibrill (Arndt et al. 2010, Hishiya et al. 2010). It is also plausible that the mutation alters the degradation and/or autophagic processes associated with the improper BAG3 protein folding as it was observed for filamin (Arndt et al. 2010).

Summarizing, our data provide additional evidence that Pro209Leu *BAG3* mutation leads to the severe childhood-onset phenotype. It is characterized not only by myofibrillar myopathy, polyneuropathy and restrictive cardiomyopathy but LQT as well. We postulate that LQT can be a part of clinical spectrum of Pro209Leu *BAG3* mutation.
